# ChART回顾性数据库局部进展期胸腺瘤的术前诱导治疗效果分析

**DOI:** 10.3779/j.issn.1009-3419.2016.07.06

**Published:** 2016-07-20

**Authors:** 煜程 魏, 志涛 谷, 毅 沈, 剑华 傅, 黎杰 谭, 鹏 张, 泳涛 韩, 椿 陈, 仁泉 张, 印 李, 克能 陈, 和忠 陈, 永煜 刘, 有斌 崔, 允 王, 烈文 庞, 振涛 于, 鑫明 周, 阳春 柳, 媛 刘, 文涛 方, 中国胸腺肿瘤协作组成员

**Affiliations:** 1 266001 青岛，青岛大学医学院附属医院胸外科 Department of Thoracic Surgery, Afliated Hospital of Qingdao University, Qingdao 266001, China; 2 200030 上海，上海交通大学附属上海胸科医院 Department of Thoracic Surgery, Shanghai Chest Hospital, Shanghai Jiao Tong University, Shanghai 200030, China; 3 510060 广州，中山大学附属肿瘤医院胸外科 Department of Thoracic Surgery, Guangdong Esophageal Cancer Institute, Sun Yat-sen University Cancer Center, State Key Laboratory of Oncology in South China, Collaborative Innovation Center of Cancer Medicine, Guangzhou 510060, China; 4 200032 上海，复旦大学附属中山医院胸外科 Department of Thoracic Surgery, Zhongshan Hospital, Fudan University, Shanghai 200032, China; 5 300052 天津，天津医科大学附属总医院胸外科 Department of Endocrinology, Tianjin Medical University General Hospital, Tianjin 300052, China; 6 610041 成都，四川省肿瘤医院胸外科 Department of Thoracic Surgery, Sichuan Cancer Hospital, Chengdu 610041, China; 7 350001 福州，福建医科大学附属协和医院胸外科 Department of Thoracic Surgery, Fujian Medical University Union Hospital, Fuzhou 350001, China; 8 230022 合肥，安徽医科大学附属第一医院胸外科 Department of Thoracic Surgery, First Afliated Hospital of Anhui Medical University, Hefei 230022, China; 9 450008 郑州，郑州大学附属肿瘤医院胸外科 Department of Thoracic Surgery, Afliated Cancer Hospital of Zhengzhou University, Zhengzhou 450008, China; 10 100142 北京，北京大学附肿瘤医院胸外科 Department of Thoracic Surgery, Beijing Cancer Hospital, Beijing 100142, China; 11 200433 上海，长海医院胸心外科 Department of Cardiothoracic Surgery, Changhai Hospital, Shanghai 200433, China; 12 110042 沈阳，辽宁肿瘤医院胸外科 Department of Thoracic Surgery, Liaoning Cancer Hospital, Shenyang 110042, China; 13 130021 长春，吉林大学附属第一医院胸外科 Department of Thoracic Surgery, First Afliated Hospital of Jilin University, Changchun 130021, China; 14 610041 成都，四川大学华西医院胸外科 Department of Thoracic Surgery, West China Hospital, Sichuan University, Chengdu 610041, China; 15 200032 上海，复旦大学附属华山医院胸外科 Department of Thoracic Surgery, Huashan Hospital, Fudan University, Shanghai 200032, China; 16 300060 天津，天津医科大学附属肿瘤医院食管癌中心 Department of Esophageal Cancer, Tianjin Cancer Hospital, Tianjin 300060, China; 17 310022 杭州，浙江省肿瘤医院胸外科 Department of Thoracic Surgery, Zhejiang Cancer Hospital, Hangzhou 310022, China; 18 330006 南昌，江西省人民医院胸外科 Department of Thoracic Surgery, Jiangxi People's Hospital, Nanchang 330006, China

**Keywords:** 局部进展, 胸腺瘤, 诱导治疗, 手术, 生存率, Local progression, Thymic malignancy, Induction therapy, Surgery, Survival

## Abstract

**背景与目的:**

探讨术前诱导治疗在胸腺瘤中的应用及其对局部进展期胸腺瘤预后的影响。

**方法:**

收集中国胸腺肿瘤协作组（Chinese Alliance of Research for Thymomas, ChART）1994年1月1日至2012年12月31日回顾性数据库中局部进展期胸腺瘤（Masaoka-Koga分期为Ⅲ期-Ⅳa期）病例。分为诱导治疗组和直接手术组，对比分析两组的R0切除率、5年复发率及5年生存率等指标。诱导治疗组术后分期为Masaoka-Koga Ⅰ期-Ⅱ期的病例视为诱导治疗后降期。为更加精确评估诱导治疗效果，在剔除术后Ⅳ期病例的基础上，再次将诱导治疗组术后Masaoka-Koga Ⅰ期-Ⅲ期的病例与直接手术组Masaoka-Koga Ⅲ期的病例进行对比分析。

**结果:**

ChART回顾性数据库1, 713例有效病例中，局部进展期胸腺瘤706例，仅68例（4%）作了术前诱导治疗，R0切除率为67.6%，5年复发率为44.9%，5年与10年生存率分别为49.7%和19.9%。其中17例诱导治疗后达到降期，降期亚组中胸腺瘤的比例高于胸腺癌（38.7% *vs* 13.9%, *P*=0.02）；与未降期亚组相比，降期亚组获得更高的5年生存率（93.8% *vs* 35.6%, *P*=0.013）。剔除术后Ⅳ期的病例后，直接手术组和诱导治疗组R0切除率接近（76.4% *vs* 73.3%, *P*=0.63），但5年生存率差异明显（85.2% *vs* 68.1%, *P* < 0.001），对于降期亚组，5年生存率优于直接手术组（93.8% *vs* 85.2%, *P*=0.438），未降期亚组5年生存率仅35.6%，明显差于降期亚组和直接手术组（*P* < 0.001）。

**结论:**

术前诱导治疗目前尚未在局部进展期胸腺瘤中广泛应用，但ChART的回顾性数据研究显示通过有效的术前诱导治疗可以使难以彻底切除的病例降期后增加R0切除的机会，从而延长生存，特别是胸腺瘤的病例。这一初步结果将有助于未来的研究。

## Abstract (en)

**Background and objective:**

To evaluate the role of preoperative induction therapy on prognosis of local-ly advanced thymic malignancies.

**Methods:**

Between 1994 and 2012, patients received preoperative induction therapies (IT group) in the Chinese Alliance for Research in Thymomas (ChART) database, were compared with those having surgery directly afer preoperative evaluation (DS group). All tumors receiving induction therapies were locally advanced (clinically stage Ⅲ-Ⅳ) before treatment and those turned out to be in pathological stage Ⅰ and Ⅱ were considered downstaged by induction. Clinical pathological characteristics were retrospectively analyzed. To more accurately study the e?ect of induction therapies, stage Ⅳ patients were then excluded. Only stage Ⅰ-Ⅲ tumors in the IT group and stage Ⅲ cases in the DS group were selected for further comparison in a subgroup analysis.

**Results:**

Only 68 (4%) out of 1, 713 patients had induction therapies, with a R0 resection of 67.6%, 5-year recurrence of 44.9%, and 5- and 10-year overall survivals (OS) of 49.7% and 19.9%. Seventeen patients (25%) were downstaged afer induction. Signifcantly more thymomas were downstaged than thymic carcinomas (38.7% *vs* 13.9%, *P*=0.02). Tumors downstaged afer induction had signifcantly higher 5-year OS than those not downstaged (93.8% *vs* 35.6%, *P*=0.013). For the subgroup analysis when stage Ⅳ patients were excluded, 5-year OS was 85.2% in the DS group and 68.1% in the IT group (*P* < 0.001), although R0 resection were similar (76.4% *vs* 73.3%, *P*=0.63). However, 5-year OS in tumors downstaged afer induction (93.8%) was similar to those in the DS group (85.2%, *P*=0.438), both signifcantly higher than those not downstaged afer induction (35.6%, *P* < 0.001).

目前为止手术切除是胸腺瘤治疗的主要方法，而R0切除和治疗前Masaoka分期以及世界卫生组织（World Health Organization, WHO）组织学分型是明确的胸腺瘤预后影响因素。但对于局部进展期胸腺瘤（Masaoka-Koga Ⅲ期-Ⅳa期），完全切除手术技术上具有一定的挑战性。尽管上腔静脉重建及全胸膜肺切除等复杂技术在很多中心已经熟练运用，但仍有相当多的病例难以达到R0切除，导致术后早期复发。因此，局部进展期胸腺瘤的治疗模式存在争议。国外若干中心开展了术前诱导治疗，以期提高局部进展期胸腺瘤的R0切除率，延长生存，取得了一定效果。由于胸腺瘤发病率较低、单中心病例有限，术前诱导治疗效果尚需更多国家、更多中心的数据积累，由此，我们分析了中国胸腺肿瘤协作组（Chinese Alliance of Research for Thymomas, ChART）回顾性数据库中的相关病例，现报告如下。

## 1 材料与方法

ChART回顾性数据库中，18家医院1994年1月1日-2012年12月31胸腺瘤病例2, 104例。删除：有无诱导治疗不明确202例；仅行活检及临床切除状态不明确45例；分期不明确25例、分型不明确108例。分期和分型明确、手术为主要治疗手段的胸腺肿瘤病例共1, 713例。其中局部进展期胸腺瘤706例，诱导治疗组（术前诱导治疗后再手术）68例，直接手术组638例。诱导化疗的方案：①CAP方案，顺铂（Cisplatin）+阿霉素（Doxorubicin）+环磷酰胺（Cyclophosphamide）；②.PE方案，顺铂（Cisplatin）+依托泊苷（etoposide）。③.Carboplatin(卡铂）+Paclitaxel（紫杉醇）方案。化疗+放疗为同步或序贯进行。

诱导治疗组病例术前评估均无法完整切除，临床分期为Masaoka-Koga Ⅲ期-Ⅳa期，术后分期变更为Masaoka-Koga Ⅰ期-Ⅱ期视为诱导治疗降期。为更加精确分析术前诱导治疗效果，剔除Masaoka-Koga Ⅳa期病例，将术后Ⅰ期-Ⅲ期的诱导治疗组病例与直接手术组Ⅲ期的病例再次进行对比分析。

统计学处理采用SPSS18.0软件进行。双侧*P* < 0.05为差异具有统计学意义。针对不同变量，采用Mann-Whitney U检验，*t*检验，卡方检验和*Fisher*精确概率法。通过*Kaplan-Meier*法建立生存曲线，两组间差异比较采用*Log-rank*检验。

## 2 结果

ChART回顾性数据库接受手术治疗1, 713例有效病例中，仅68例接受术前诱导治疗，占所有病例4%。其中单纯化疗30例，单纯放疗9例，化疗+放疗29例。早期（1994年-2003年）与近期（2004年-2012年）的病例中接受诱导治疗的比例无差异（3.8% *vs* 4.9%, *P*=0.458，表 1），但诱导治疗的模式有所变化，近期化疗+放疗的病例减少，单独化疗或单独放疗的病例增加（图 1）。

**1:** 早期与近期两个阶段接受术前诱导治疗的比例（1994年-2003年及2004年-2012年） Incidence rate of preoperation induction therapy, and the tendency of receiving whether induction therapy or operation directly in two periods, 1994-2003 and 2004-2012

		Induction therapy		Total
		No	Yes	
Treatment period	2004-2012	1, 430(96.2%)	57(3.8%)	1, 487(100%)
	1994-2003	215(95.1%)	11(4.9%)	226(100%)
Total		1, 645(96.0%)	68(4.0%)	1, 713(100%)
注：本表得到版权所有者©2011-2016 Journal of Thoracic Disease复制许可。				

**1:**
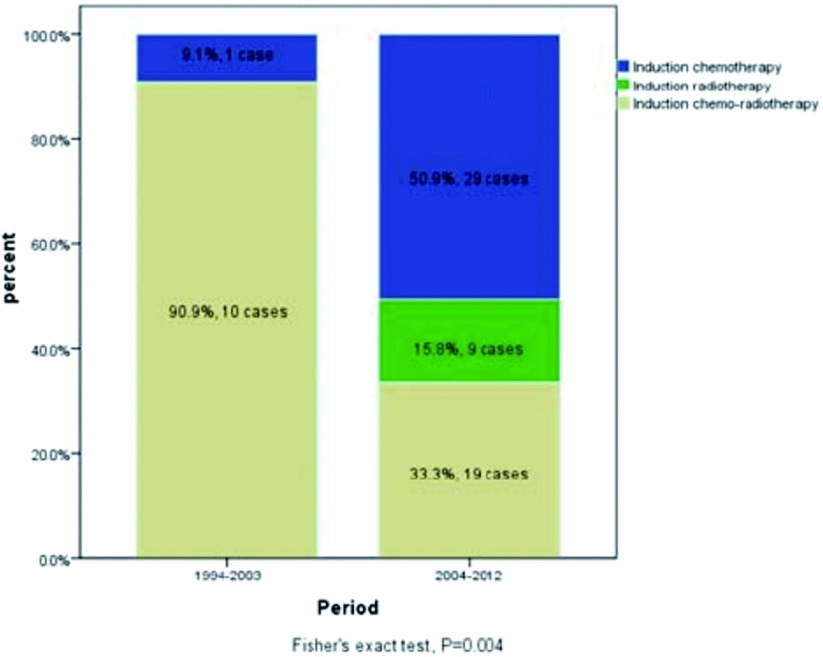
早期与近期两个阶段术前诱导治疗模式的变化（1994年-2003年及2004年-2012年） Variation of different induction therapy in two periods, 1994-2003 and 2004-2012

诱导治疗组中男43例、女25例，平均年龄（44.8±14.9）岁，合并重症肌无力5例（7.4%）。肿瘤平均最大径为（8.1±2.9）cm。R0切除率为67.6%，术后分期证实诱导治疗后降期为Ⅰ期9例（13.2%）、Ⅱ期8例（11.8%），51例诱导治疗后没有降期，其中Ⅲ期38例（55.9%）、Ⅳ期13例(19.1%)。WHO病理分型：A型2例（2.9%）、AB型5例（7.4%）、B1型5例（7.4%）、B2型8例（11.8%）、B3型12例（17.6%）、胸腺癌34例（50%）、胸腺类癌2例（2.9%）。5年与10年生存率分别为49.7%与19.9%，5年累积复发率为44.9%（图 2，图 3）。诱导治疗后胸腺瘤降期的比例明显高于胸腺癌与胸腺类癌（38.% *vs* 13.9%, *P*=0.02）。R0切除的病例5年生存率高于R1和R2切除的病例，但统计学无差异（58.2% *vs* 19.6%, *P*=0.134，图 4）。另一方面，诱导治疗后降期亚组5年生存率高于未降期亚组（93.8% *vs* 35.6%，*P*=0.134，图 5）。

**2:**
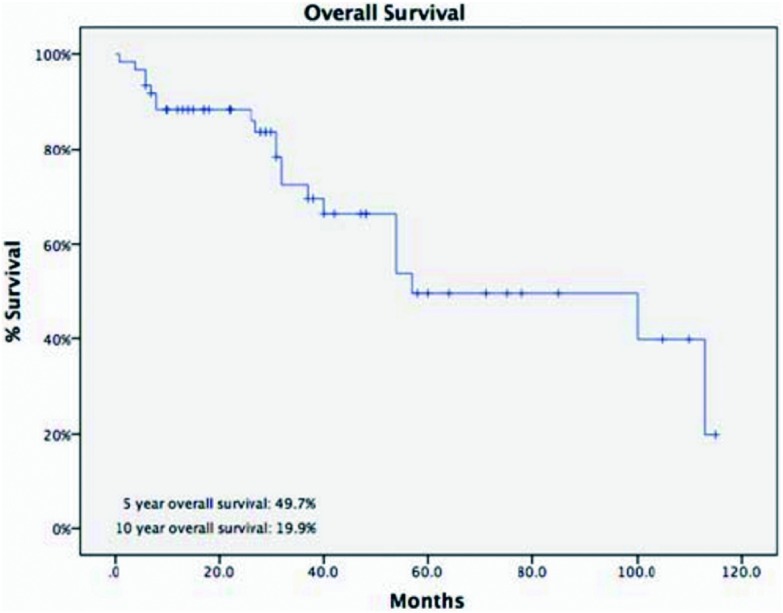
诱导治疗组的5年生存率 The 5-year overall survival rate of patients in induction therapy group

**3:**
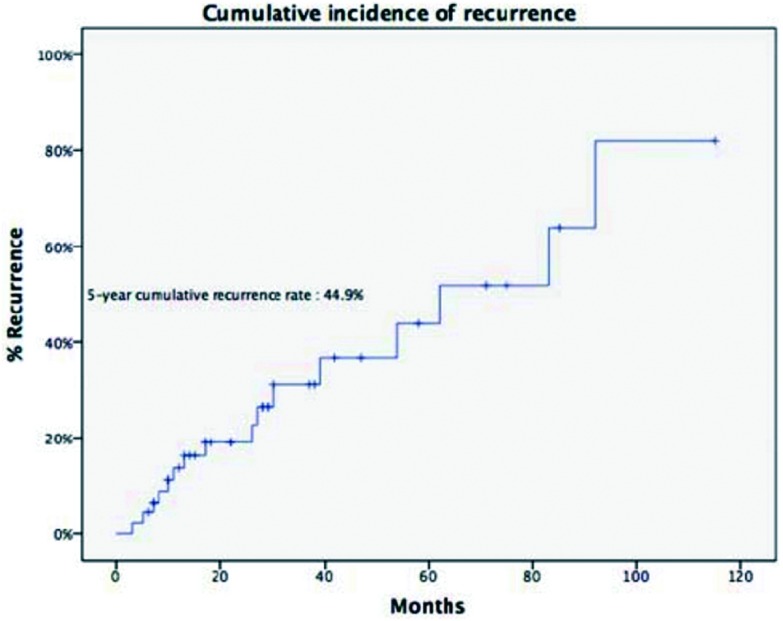
诱导治疗组的5年累积复发率 The 5-year recurrence rate of patients in induction therapy group

**4:**
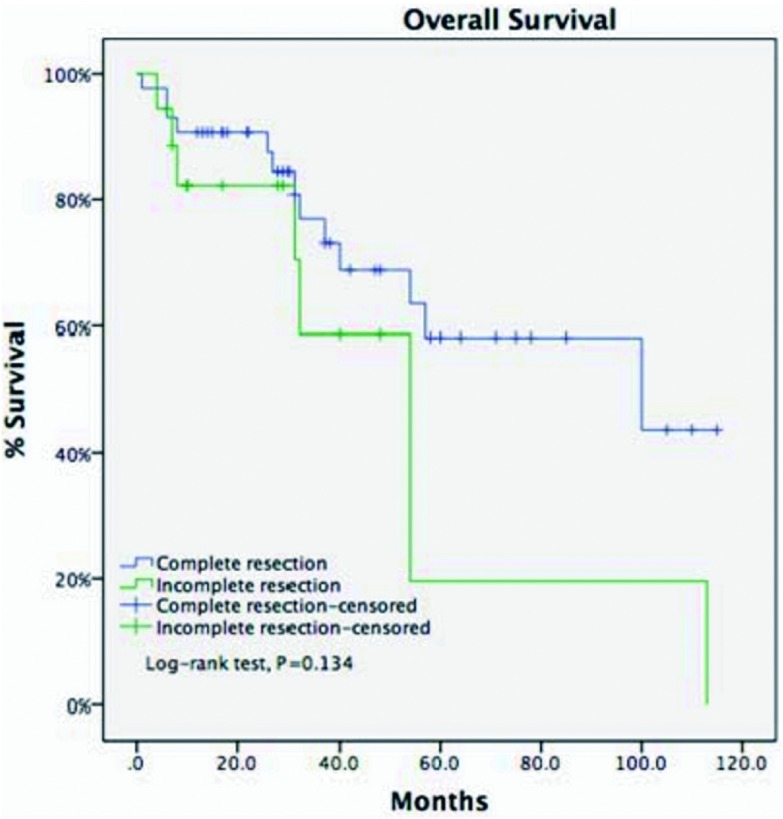
诱导治疗组完全切除病例的5年生存率（R0, 58.2%）高于未完全切除的病例（R1+R2, 19.6%），但无统计学差异（*P*=0.134）。 Five-year overall survival in patients who had their tumors resected completely (R0, 58.2%) was higher than those with incomplete resections (R1 and R2, 19.6%). But the difference did not reach statistical significance (*P*=0.134).

**5:**
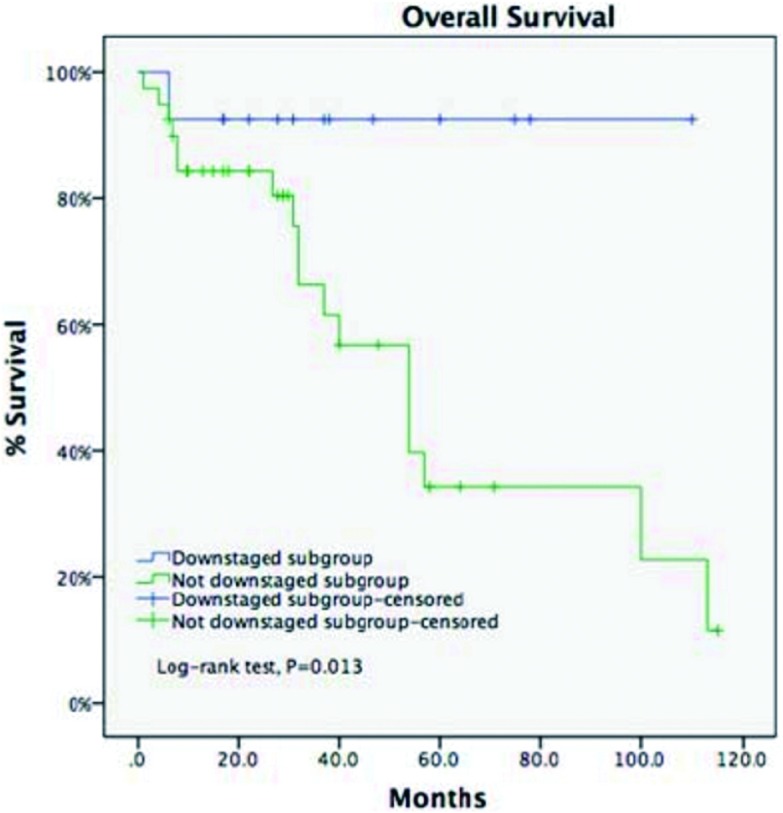
诱导治疗组中降期亚组与未降期亚组5年生存率比较（93.8% *vs* 35.6%, *P*=0.013) For the IT group, downstaging subgroup got a longer 5-year OS compared with undownstaging subgroup (93.8% *vs* 35.6%, *P*=0.013)

剔除Masaoka-Koga Ⅳ期病例，诱导治疗组非Ⅳ期病例（包含17例，30.9%诱导治疗后降期至Ⅰ期-Ⅱ期的病例）与直接手术组Ⅲ期病例进行比较。两组基线特征大多近似，诱导治疗组合并重症肌无力病例比例更低，而胸腺癌与胸腺类癌病例比例更高（表 2）。两组的平均肿瘤最大径相近，R0切除率无差异（76.4% *vs* 73.3%, *P*=0.63），5年生存率直接手术组优于诱导治疗组（85.2% *vs* 68.1%, *P* < 0.001，图 6）。5年累计复发率诱导治疗组高于直接手术组（58% *vs* 23%, *P* < 0.001，图 7）。诱导治疗后降期亚组的5年生存率与直接手术组相近（93.8% *vs* 85.2%, *P*=0.438），均优于诱导治疗后未降期亚组（35.6%，*P* < 0.001，图 8）。按胸腺瘤及胸腺癌分层分析（剔除Ⅳ期病例），诱导治疗后降期亚组均可见生存获益：胸腺瘤病例，诱导治疗后降期亚组、直接手术组与诱导治疗后未降期亚组5年生存率分别为100%、91.1%和39.6%（*P* < 0.001）；胸腺癌病例3组的5年生存率分别为80%、70.6%和24.4%（*P*=0.182），降期亚组和未降期亚组相比无统计学差异（*P*=0.517）。

**2:** 诱导治疗组和直接手术组临床病理特征(剔除Masaoka-Koga Ⅳ期病例) Patients' characteristics of Masaoka-Koga stage Ⅲ in the DS group and those of pathological Masaoka-Koga stage Ⅰ-Ⅲ in the IT group

Variables	IT(*n*=55)	DS(*n*=499)	*P* value
Gender			0.941
Male	34(61.8%)	311(62.3%)	
Female	21(38.2%)	188(37.7%)	
Age(yr, mean±SD)	45.3±14.7	51.6±13	0.135
Tumor size(cm, mean±SD)	7.96±2.7	7.92±3.2	0.224
Preoperative MG			0.000
No	50(90.9%)	379(76%)	
Yes	5(9.1%)	120(24%)	
WHO histological types			0.332
A	2(3.6%)	10(2%)	
AB	5(9.1%)	40(8%)	
B1	4(7.3%)	40(8%)	
B2	4(7.3%)	40(8%)	
B3	11(20%)	140(28.1%)	
C	28(50.9%)	183(36.7%)	
NETT	1(1.8%)	16(3.2%)	
WHO histological types(combination)			0.022
Thymoma	26(47.3%)	300(60.1%)	
C+NETT	29(52.7%)	199(39.9%)	
Resection State			0.63
R0	42(76.4%)	366(73.3%)	
R1+R2	13(23.6%)	133(26.7%)	
IT: Induction therapies; DS: Directly surgery; C: Carcinoma; NETT: Neuroendocrine thymic tumor. 注：本表得到版权所有者©2011-2016 Journal of Thoracic Disease复制许可。			

**6:**
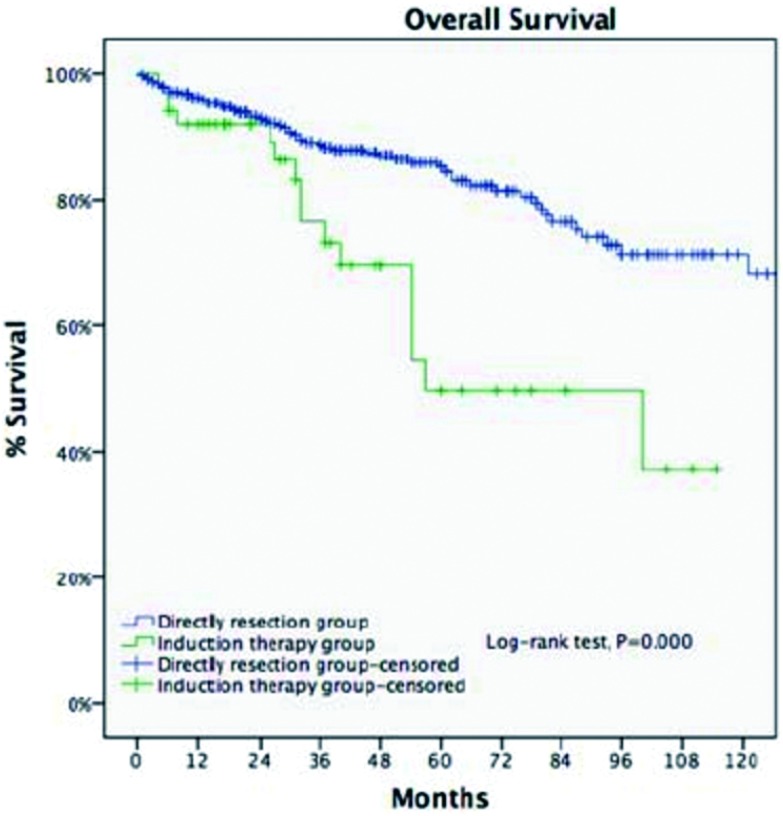
剔除Ⅳ期病例后直接手术组与诱导治疗组5年生存率比较(85.2% *vs* 68.1%, *P* < 0.001)。 Ten-year OS of Masaoka-Koga staging Ⅲ patients in the DS group was significantly higher than patients of pathological Masaoka-Koga stage Ⅰ-Ⅲ in the IT group (85.2% *vs* 68.1%, *P* < 0.001).

**7:**
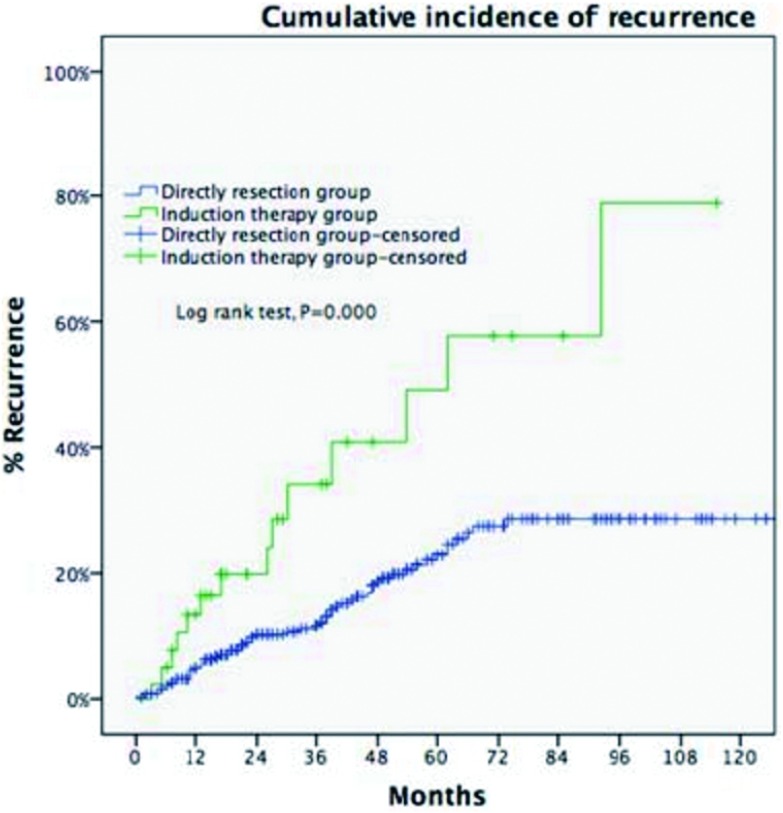
累积5年复发率直接手术组（Masaoka-Koga staging Ⅲ期）低于诱导治疗组（Masaoka-Koga stage Ⅰ-Ⅲ）：23% *vs* 58%, *P* < 0.001。 Five-year CIR of Masaoka-Koga staging Ⅲ patients in the DS group was significantly lower than patients of pathological Masaoka-Koga stage Ⅰ-Ⅲ in the IT group (23% *vs* 58%, *P* < 0.001).

**8:**
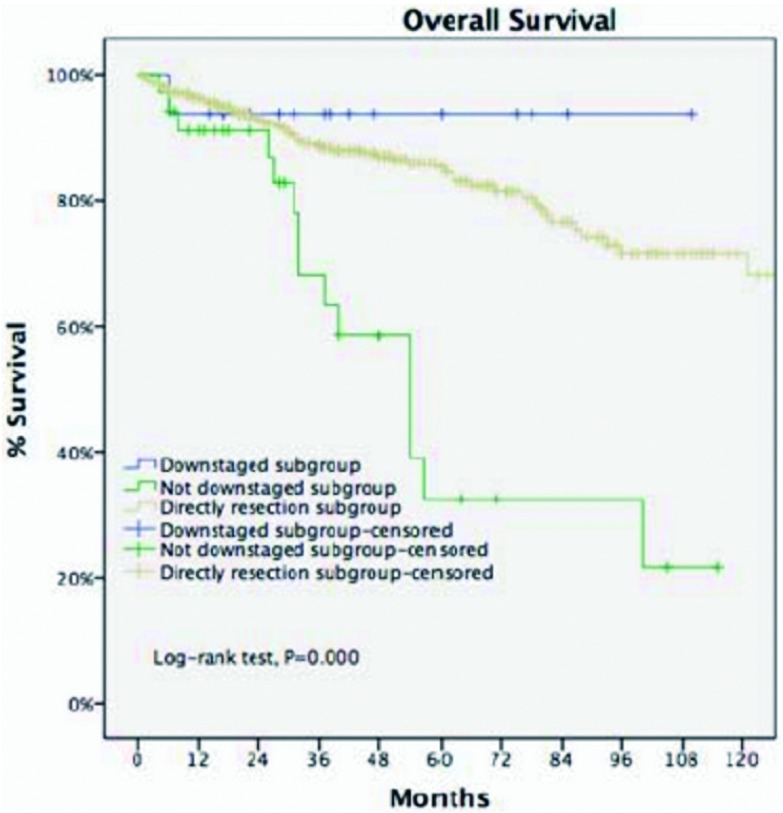
诱导治疗后降期亚组5年生存率与直接手术组接近（93.8% *vs* 85.2%, *P*=0.438）, 两组均优于诱导治疗后未降期亚组（*P* < 0.001）。 For locally advanced thymic malignancies, 5-year OS of downstage patients after induction therapy in IT group was similar to those in the DS group (93.8% *vs* 85.2%, *P*=0.438), both significantly greater than undownstage subgroup (*P* < 0.001).

## 3 讨论

胸腺肿瘤的预后取决于肿瘤分期、组织学分型和手术切除的完整性^[1-3]^。前两个因素患者就诊时已固定不变，因此能否完整切除成为决定性因素。对于局部进展期胸腺瘤病例，即使应用扩大切除等技术，完整切除仍然常常难以做到。我们的资料显示，即使做了术前诱导治疗，R0切除率也仅有67.6%。但术前诱导治疗在人体其他常见实体恶性肿瘤治疗中的作用已经证实有效，原因在于：①诱导治疗后可使肿瘤的分期降期，从而增加完整切除的机会；②通过早期系统治疗控制肿瘤发展；③防止术中肿瘤播散^[4]^。然而目前为止，有关局部进展期胸腺瘤的术前诱导治疗缺乏前瞻性随机对照研究，仅有为数不多的小样本回顾性研究报道^[5]^。ChART回顾性数据库收集例1, 713例资料完整的病例，也仅有68例实施了术前诱导治疗。

目前文献报道的样本量最大的回顾性研究纳入了63例病例。33例应用诱导治疗（放疗8例，化疗25例）、30例直接手术^[6]^。两组Ⅲ期病例R0切除率为65% *vs* 46%，Ⅳ期病例R0切除率为0 *vs* 20%。诱导治疗组的无疾病生存（progression free survival, PFS）略低于直接手术组，但5年生存率无差异。该研究的诱导治疗后R0切除率与Ch ART回顾性数据库诱导治疗后67.6%的R0切除率相近。另外一个较大样本的单中心报道纳入61例，统计分析结果完整切除、Masaoka分期、WHO组织分型和诱导治疗为独立预后影响因素^[7]^。

ChART数据库显示，过去20年仅有4%的病例接受术前诱导治疗，原因在于缺乏前瞻性对照研究，无法建立共识。此外单纯化疗或放疗的病例增加，联合放化疗的病例减少，手术难度的增加及术后管理困难是潜在的原因。另一方面，这一数据库中诱导治疗组仅有25%的病例达到降期，胸腺瘤降期病例多于胸腺癌降期病例（38.7% *vs* 13.9%, *P*=0.02）；完整切除病例5年生存率优于非完整切除病例（58.2% *vs* 19.6%），由于诱导治疗组样本量远少于直接手术组，差异未显示有统计学意义；但诱导治疗后降期亚组的5年生存率优于未降期亚组（93.8% *vs* 35.6%, *P*=0.013）。由此，进展期胸腺瘤诱导治疗前瞻性研究势在必行，另一方面有效的诱导治疗方案亟待开发。

由于Ⅳ期病例更难完整切除，我们将两组的Ⅳ期病例剔除，再次分析统计，以利于更加精确评估诱导治疗的效果。两组的R0切除率接近（76.4% *vs* 73.3%，*P*=0.63，诱导治疗组在前），但5年累积复发率及5年生存率，诱导治疗组差于直接手术组。可能的因素为接受诱导治疗的病例本身浸润及外侵更重、直接手术更难以完整切除，另外，诱导治疗组包含更多的胸腺癌病例，而胸腺癌的恶性程度远高于胸腺瘤。一个值得注意的发现是，胸腺瘤对诱导治疗的敏感性优于胸腺癌，提示我们在未来的研究中，针对胸腺瘤和胸腺癌诱导治疗可能应采用不同的方法。

诱导治疗亚组分析，降期亚组5年生存率为93.8%，直接手术组为85.2%，均优于未降期亚组（35.6%, *P* < 0.001）。根据组织学类型（胸腺瘤和胸腺癌）进行分层分析，诱导治疗降期后不仅胸腺瘤有生存获益，胸腺癌同样得到生存获益（降期亚组5年OS为80%，未降期亚组24.4%，*P*=0.517）。由于各组间样本量不均衡（诱导治疗组胸腺癌仅29例），统计学结果难以得到极具说服力的结果，但分层后可见两种病理类型的肿瘤诱导治疗降期后均可得到生存获益，未降期则反之。提示：术前评估不可完整切除的病例，如诱导治疗未降期，再选择手术的价值值得商榷，这种情况下根治性放化疗可能为最佳选择。

由于回顾性研究的限制，究竟哪一种诱导治疗方案更好尚无法定论，ChART数据库的数据也是如此。文献报道的传统的几种化疗方法ChART数据库的病例均涉及，总的客观反应率为25%-90%^[8-10]^。日本一项二期临床试验（JCOG 9606）对传统化疗方案进行改变，采用剂量密集的周化疗方案，连续化疗9周，然后再进行手术^[11]^，客观反应率为62%。此外，糖皮质激素有减少淋巴细胞的效应，因此，可使含淋巴细胞的B型和AB型胸腺瘤缩小^[12, 13]^，利于完整切除，故上述化疗方案在应用中常加入糖皮质激素。但文献报道，糖皮质激素虽然增加化疗的缓解率，然而对长期生存率并无影响。由于铂类和紫杉类化疗药物可增加肿瘤对放疗的敏感性，因此联合放化疗肿瘤的反应率可能会更高、降期的可能更大^[14]^。

另一方面，随着分子靶向药物治疗的研究进展，胸腺瘤靶向治疗也逐步展开^[15-17]^。相关癌基因的信号转导通路可能是胸腺瘤靶向治疗方向^[18]^，包括表皮生长因子受体（epidermal growth factor receptor, EGFR），肥大/干细胞生长因子受体（mast/stem-cell growth factor receptor），类胰岛素生长因子1受体（insulin-like growth factor 1 receptor, IGF-1R）等等。目前，国外正进行一项有关进展期胸腺瘤的化疗结合西妥昔单抗诱导治疗后再手术的代号为NCT01025089的前瞻性研究^[5]^。这一方向的研究有望为进展期胸腺瘤的治疗带来新的曙光。

## 4 结论

由于胸腺瘤发病率较低，目前尚无进展期胸腺瘤术前诱导治疗的前瞻性随机对照研究。我们分析的Ch A RT回顾性数据库是近20年18家中心的病例积累。得出如下结论：①尽管诱导治疗的模式仍有待探讨，对于初诊时不能完全切除或完全切除可能性不确定的局部进展期胸腺瘤病例，术前诱导治疗有获益的可能；②诱导治疗反应好的病例可提高长期生存率；③对诱导治疗反应差的病例，即使随后接受手术治疗，生存获益有限；④胸腺瘤与胸腺癌有显著不同的临床特点，对诱导治疗的反应也不同。这些初步结论有助于未来该领域的进一步深入研究。
